# Baicalin mitigated *Mycoplasma gallisepticum*-induced structural damage and attenuated oxidative stress and apoptosis in chicken thymus through the Nrf2/HO-1 defence pathway

**DOI:** 10.1186/s13567-019-0703-6

**Published:** 2019-10-21

**Authors:** Jichang Li, Zujian Qiao, Wanying Hu, Wei Zhang, Syed Waqas Ali Shah, Muhammad Ishfaq

**Affiliations:** 1 Heilongjiang Key Laboratory for Animal Disease Control and Pharmaceutical Development College of Veterinary Medicine, Northeast Agricultural University, 600 Changjiang Road, Xiangfang District, Harbin, 150030 China; 2 College of Animal Science and Technology, Northeast Agricultural University, Harbin, China; 3 Harbin Veterinary Research Institute, Chinese Academy of Agricultural Sciences, 678 Haping Road, Xiangfang District, Harbin, 150086 China

## Abstract

The thymus is a primary lymphoid organ and plays a critical role in the immune response against infectious agents. Baicalin is a naturally derived flavonoid famous for its pharmacological properties, but the preventive effects of baicalin against immune impairment remain unclear. We examined this effect in the context of *Mycoplasma gallisepticum* (MG) infection-induced structural damage in the chicken thymus. Histopathological examination showed that the compact arrangement of cells in the thymus was lost in the MG-infected group. Inflammatory cell infiltration and nuclear debris accumulated, and the boundary between the cortex and medulla was not clearly visible. The mRNA and protein expression of apoptosis-related genes were significantly increased in the MG-infected group compared to the control group and the baicalin group. The number of positively stained nuclei in the terminal deoxynucleotidyl transferase-mediated dUTP nick end labelling (TUNEL) assay were increased in the MG-infected group. In addition, electron microscopic examination showed chromatin condensation, mitochondrial swelling and apoptotic vesicles in the MG-infected group. However, baicalin treatment significantly alleviated the oxidative stress and apoptosis induced by MG infection. Importantly, the abnormal morphology was partially ameliorated by baicalin treatment. Compared to the MG-infected group, the baicalin-treated group showed significantly reduced expression of apoptosis-related genes at both the mRNA and protein levels. Meanwhile, the nuclear factor erythroid 2-related factor 2 (Nrf2) signalling pathway and downstream genes were significantly upregulated by baicalin to counteract MG-induced oxidative stress and apoptosis in the thymocytes of chickens. In summary, these findings suggest that baicalin treatment efficiently attenuated oxidative stress and apoptosis by activating the Nrf2 signalling pathway and could protect the thymus from MG infection-mediated structural and functional damage.

## Introduction

*Mycoplasma gallisepticum* (MG) are wall-less prokaryotic microorganisms that belong to the family *Mycoplasmataceae* and class *Mollicutes* [1]. MG is the primary aetiologic agent of infectious sinusitis in game birds, turkeys, passerine birds and pigeons of all ages and chronic respiratory disease in chickens [2]. This pathogen is highly virulent and causes major economic losses in the poultry industry worldwide. It has been reported that MG infection causes immune dysregulation in poultry [3], but the underlying mechanism is still unclear. It has been previously demonstrated that MG infection causes atrophy in chicken lymphoid organs such as the thymus, bursa and spleen [4]. Studies on the thymus are especially important in infectious disease immunology because the thymus is a primary lymphoid organ and a site for the maturation of T cells that finally colonize secondary lymphoid organs to fight against invading pathogens [5]. Theoretically, thymic injury can cause immune impairment and results in serious consequences associated with the development of an immature immune system, which are associated with tissue homeostasis [6]. Fascinatingly, birds lacking a thymus are unable to clear MG infection, and increased lesions were observed in these birds compared to normal birds [7]. These findings showed that a fully functional humoural and cellular immune system is essential to eliminate MG infections. However, the effect of MG infection on thymus function is still elusive and needs to be studied.

Previous studies demonstrated that MG infection causes respiratory distress and chronic infection mainly by the colonization of the host cells [8]. Moreover, persistent and increased oxidative stress induced immune impairment through cellular DNA damage and biomolecule fragmentation [9–11]. The complex interaction between cells inflammatory cytokines/chemokines is associated with disease outcome. However, immunoregulatory and inflammatory cytokines are involved in the stimulation of leukocytes, in which promotes bacterial clearance [12]. The dysregulation of these molecules, including but not limited to interleukin-6 (IL-6), IL-1β, IL-8, IL-10, gamma interferon (IFN-γ), and tumor necrosis factor alpha (TNF-α), has been found to play a critical role in mycoplasma disease pathogenesis [8]. In addition, MG produces oxidative stress, which further exacerbates the inflammatory response, and both oxidative stress and inflammation are crucial factors that can induce apoptosis [2–4]. For instance, it was suggested earlier that the severity of clinical disease is proportional to the level of apoptosis [13]. However, further research is needed to investigate the mechanism of MG-induced oxidative stress and apoptosis in the thymus of chickens. Previous reports demonstrated that autophagy plays a critical role in the cell homeostasis mechanism associated with many pathological and physiological conditions [14]. Autophagy protects cells from invading pathogens and clears invading microbes [15]. Our previous study showed that MG induced autophagy through the ERK signalling pathway in a process mediated by toll-like receptor 2 (TLR2) in RAW264.7 cells [16]. Hence, the induction of autophagy by natural or chemical drugs could be a promising strategy to prevent MG infection.

Baicalin is derived from the plant *Scutellaria baicalensis* Georgi belonging to the family *Lamiaceae*. It is famous in many countries, including the Russian Federation, European countries and several East Asian countries, for its potential pharmacological properties [17]. The chemopreventive properties of baicalin have been attributed to its multifold pharmacological effects, including anti-tumour effect, anti-inflammation effect, apoptosis modulation, autophagy induction, cell cycle arrest, metastasis suppression and oxidative stress inhibition [18]. Recently, studies reported that baicalin can upregulate the transcription factor nuclear factor erythroid 2-related factor 2 (Nrf2) signalling pathway [19, 20]. Nrf2 was reported to be responsible for cytoprotection and to stimulate a number of antioxidant and detoxifying enzymes, including NAD(P)H:quinone oxidoreductase-1 (NQO-1), glutathione S-transferase (GST) and heme-oxygenase-1 (HO-1) [21]. However, the protective role of baicalin against MG infection-mediated oxidative stress and apoptosis in the chicken thymus has not been reported. The main objectives of the present study were to investigate the protective properties of baicalin against MG-induced oxidative stress and apoptosis in the chicken thymus. We noted that baicalin is associated with Nrf2 upregulation at the molecular level and activated antioxidant enzymes. The complex interplay among oxidative stress, inflammation-related cytokines, apoptosis and the Nrf2 signalling pathway was the major mediator of baicalin-mediated protection.

## Materials and methods

### Reagents and chemicals

Baicalin (purity ≥ 98.0%) was provided by Huifeng Animal Health Co., Ltd. (Harbin, Heilongjiang, China). The detection kits for antioxidant parameters CAT, SOD, MDA, GSH and γ-GT were purchased from the Nanjing Jiancheng Institute of Biotechnology (Nanjing, China). TRIzol reagent was obtained from Life Science Technologies Corporation (Cal., USA). Gibco BRL (Gaithersburg, MD, USA) provided foetal bovine serum (FBS). Eosin, and haematoxylin dye was purchased from Nanjing Chemical Reagent Factory (Nanjing, China). Isopropanol, methanol, glycine, ethanol (99.0) and chloroform were provided by Kermel Chemical Reagent Company (Tianjin, China).

### Growth of MG strain R_low_

MG strain R_low_ was obtained from Harbin Veterinary Research Institute (Heilongjiang, China), Chinese Academy of Agricultural Sciences. Modified Hayflick medium, consisting of 0.1% nicotinamide adenine dinucleotide (NAD), 0.05% penicillin, 20% FBS, 0.05% thallium acetate and 10% freshly prepared yeast extract, was used to culture MG as described in a previous study [16]. A change in colour from phenol red to orange was observed when MG reached the mid-exponential phase.

### Experimental animals and treatments

Healthy white leghorn chickens (1 day old) were obtained from Chia Chau chicken farm (Harbin, China). Chickens were adapted to experimental conditions before starting experiments. Ad libitum feed and fresh drinking water were provided to chickens. The experimental groups included the (A) control group (B) MG-infected group (C) baicalin group (450 mg/kg) and (D) MG-treated baicalin group (450 mg/kg). A previously published method [22] was used to challenge chickens with MG strain R_low_ at a density of 1 × 10^9^ CCU/mL. Baicalin treatment was started after 3 days of MG infection and given orally [23] once per day for seven consecutive days. Chickens were sacrificed after 7 days of baicalin treatment, and thymus samples were collected for further analyses.

### Determination of antioxidant activities

CAT, SOD, MDA, γ-GT and GSH-Px enzyme activities were measured in thymus tissues according to the manufacturer’s instructions (Jiancheng Bioengineering Institute, Nanjing, China). Fresh samples were prepared in ninefold physiological saline at 4 °C and centrifuged (1000 × *g*) for 10 min. The supernatant was collected and used to examine the above enzyme activities.

### Cytokine activities assay

Thymus samples were first homogenized in physiological saline solution and centrifuged for 10 min at 1000 × *g*. The supernatant was collected, and TNF-α, IL-6, IL-1β and IL-8 enzyme activities were determined by ELISA according to the manufacturer’s instructions. A blank control and duplicate samples were run on an iMARKTM microplate reader (Bio-Rad Co., Ltd. Shanghai, China).

### Histopathological and ultrastructural observations

Thymus samples were prepared for histopathological examination as described previously [24]. In brief, samples were fixed in 10% neutral formalin and processed in graded ethanol. The specimens were then processed for paraffin wax, cut into sections and mounted on glass slides. Next, the slides were stained with eosin and haematoxylin dye and observed under a light microscope (Nikon E100, Tokyo, Japan). Ultrastructural examination was carried out as described previously [25], with a few modifications. Briefly, thymus samples were fixed in 2.5% glutaraldehyde and rinsed twice in 0.2 M phosphate buffer (pH = 7.2) for 15 min. Afterwards, the specimens were fixed in 1% osmium tetroxide for 1 h, dehydrated in graded ethanol and embedded in epoxy resin. Following staining with uranyl acetate, ultrathin sections were then observed under a transmission electron microscope (GEM-1200ES, JEOL Ltd., Tokyo, Japan).

### Terminal deoxynucleotidyl transferase-mediated dUTP nick end labelling (TUNEL) assay

The apoptotic cells in the thymus tissues of all experimental groups were detected by terminal deoxynucleotidyl transferase-mediated dUTP nick end labelling (TUNEL) assay. Thymus samples were first fixed in 4% formalin, processed in graded ethanol for dehydration and embedded in paraffin wax. The embedded specimens were then cut into very small sections and mounted on glass slides, and the apoptotic cells were detected by a TUNEL assay kit (Roche Diagnostics GmbH, Mannheim, USA) according to the instructions of the kit. Endogenous peroxidase activity was inhibited by hydrogen peroxide following treatment with proteinase K. The slides were incubated in the terminal TdT/nucleotide mixture for 1 h at 37 °C. Afterwards, diaminobenzidine and horseradish peroxidase were used to label nuclei, haematoxylin was used for counterstaining, and the slides were observed under a microscope (80i, Nikon Eclipse, Tokyo, Japan).

### RNA extraction and quantitative real-time polymerase chain reaction (qRT-PCR)

Total RNA was isolated from chicken thymus samples from each experimental group using TRIzol reagent. The purity and concentration of the RNA were determined spectrophotometrically by a NanoDrop (Thermo Scientific, Carlsbad, USA) at 260/280 nm [26]. The RNA samples were converted into first strand cDNA using a cDNA synthesis kit (Cat. # RR047A, Takara, Dalian, China) according to the manufacturer’s instructions. One microgram of total RNA was treated with gDNA eraser (42 °C for 2 min) to remove genomic DNA from the samples. Then, the samples (20 µL) were reverse transcribed at 37 °C for 15 min and 85 °C for 5 s in a gradient thermocycler machine (Goettingen, Germany). qRT-PCR was carried out with a kit in a 20 µL reaction volume in a Roche LightCycler instrument (Shanghai, China) according to the manufacturer’s instructions (Cat. # RR820A Takara, China). The amplification conditions are as follows. The initial denaturation was carried out at 95 °C for 30 s, followed by 2-step PCR (40 cycles: 95 °C for 5 s, and 60 °C for 45 s) and dissociation. The gene-specific primers are shown in Table 1. The β-actin gene was used as an internal reference gene. The data were analysed by the Livak and Schmittgen method [27].

**Table 1:** **List of primers used for qRT-PCR**

S. No.	Gene name	Primers (from 5′ to 3′)	Product length
1	TLR2-A	Forward 5′-TCGCTCCAACACCTTCGCATTC Reverse 5′-GATTGTCACCGTCGATCCTCAGC	181
2	TLR2-B	Forward 5′-TCCTCATCCTGGTGGTCGTTGG Reverse 5′-GGCTTCCGCTTGGCTTGGAG	88
3	TLR4	Forward 5′-TTCGGTTGGTGGACCTGAATCTTG Reverse 5′-ACAGCTTCTCAGCAGGCAATTCC	114
4	NLRP3	Forward 5′-GCTCCTTGCGTGCTCTAAGACC Reverse 5′-TTGTGCTTCCAGATGCCGTCAG	150
5	IL-10	Forward 5′-CAGCACCAGTCATCAGCAGAGC Reverse 5′-GCAGGTGAAGAAGCGGTGACAG	94
6	IL-18	Forward 5′-AGATGATGAGCTGGAATGCGATGC Reverse 5′-ATCTGGACGAACCACAAGCAACTG	97
7	PTGE	Forward 5′-GCCTTCTACAGCACGATCCTGATC Reverse 5′-GCCTTCTTCCTGAGCCTCACTTG	80
8	ALOX5	Forward 5′-GCGGTTCACAATAGCCATCAACAC Reverse 5′-GCTGTAGGTCAGGTCCTTCATTGC	139
9	ALOX15B	Forward 5′-GTGAAGGAGCGGACAGTGAAGTG Reverse 5′-AACCAGGCATCCTCCAGGAAGAG	95
10	LOXHD1	Forward 5′-CACAGACAAGACCTTCCGCTTCC Reverse 5′-GCAGTCCGTTCCTTCAGTTCCAG	123
11	IFN-γ	Forward 5′-TTCCTGATGGCGTGAAGAAGGTG Reverse 5′-TCGGAGGATCCACCAGCTTCTG	129
12	GSDMA	Forward 5′-AGCCTCACAGAAGCCATCTCCTAC Reverse 5′-GCTGCTGCTGCTCGCTGAAG	196
13	GSDME	Forward 5′-GCTGCGTGCCTGCTCTGATC Reverse 5′-GCTCAGTGCCAAGGTGCCATC	88
14	TRAF2	Forward 5′-CGTGGTGATGAAAGGACCCA Reverse 5′-AATGATGTGCTCCCGGTTGT	100
15	TNF-R1	Forward 5′-CCTGTCTGTCTTCCCTGTCC Reverse 5′-GGTGCATGGGGTCTTTTCTA	120
16	TRADD	Forward 5′-CTAGAGCCCAAAGGAAGTCGAT Reverse 5′-TGGCTGCTTCTCTGTGACAT	100
17	FADD	Forward 5′-GGGGTAAAGAGGCTGAACTCTTA Reverse 5′-TGAGTCCTATTGCACTGCTGTC	163
18	Nrf2	Forward 5′-GATGTCACCCTGCCCTTAGA-3′ Reverse 5′-TCGTTCCATTTGTTCCTTCTG-3′	124
19	GPX-1	Forward 5′-AAGTGCTGCTGGTGGTCAACG Reverse 5′-GTTGGTGGCGTTCTCCTGGTG	155
20	GPX-3	Forward 5′-GTGGCAGAGGAGTTCGGCAAC Reverse 5′-TCTTGACAGTGGCGATGTTGGC	151
21	PRDX6	Forward 5′-GCATCCGCTTCCACGACTTCC Reverse 5′-GGCGTTGATGTCCTTGCTCCAG	200
22	MAP1LC3	Forward 5′-GCTGCCAGTGCTGGACAAGAC Reverse 5′-TCCTCATCCTTCTCCTGCTCGTAG	189
23	Beclin-1	Forward 5′-ACCGCAAGATTGTGGCTGAAGAC Reverse 5′-TGAGCATAACGCATCTGGTTCTCC	163
24	mTOR	Forward 5′-AACCACTGCTCGCCACAATGC Reverse 5′-CATAGGATCGCCACACGGATTAGC	120
25	HO-1	Forward 5′-TCATTGGCAAGAAGCATCCAGAGC-3′ Reverse 5′-GAACTTGGTGGCGTTGGAGACTC-3′	176
26	NF-κB	Forward 5′-CACATGGTGGTGACCGCCAATAG-3′ Reverse 5′-GTGCCATCGTATGTAGTGCTGTCC-3′	194
27	TNF-α	Forward 5′-TGATCGTGACACGTCTCTGC-3′ Reverse 5′-CAACCAGCTATGCACCCCAG-3′	88
28	IL-6	Forward 5′-TTCACCGTGTGCGAGAACAGC-3′ Reverse 5′-CAGCCGTCCTCCTCCGTCAC-3′	80
29	IL-1β	Forward 5′-AGCAGCCTCAGCGAAGAGACC-3′ Reverse 5′-GTCCACTGTGGTGTGCTCAGAATC-3′	90
30	Bax	Forward 5′-ACTCTGCTGCTGCTCTCCTCTC-3′ Reverse 5′-ATCCACGCAGTGCCAGATGTAATC-3′	174
31	Caspase-3	Forward 5′-TACCGGACTGTCATCTCGTTCAGG-3′ Reverse 5′-ACTGCTTCGCTTGCTGTGATCTTC-3′	166
32	Caspase-8	Forward 5′-GGAAGCAGTGCCAGAACTCAGAAG-3′ Reverse 5′-TTGTTGTGGTCCATGCACCGATAG-3′	174
33	Caspase-9	Forward 5′-CCGAAGGAGCAAGCACGACAG-3′ Reverse 5′-CATCTAGCATGTCAGCCAGGTCAC-3′	121
34	P53	Forward 5′-GGAGATGGAACCATTGCTGGAACC-3′ Reverse 5′-GCTCCTGCCAGTTGCTGTGATC-3′	113
35	Bcl2	Forward 5′-GAGTTCGGCGGCGTGATGTG-3′ Reverse 5′-TTCAGGTACTCGGTCATCCAGGTG-3′	92
36	Cytochrome C	Forward 5′-CCTAATCGCCGTGGCCTTCTTAAC-3′ Reverse 5′-GGAGGAGGTAGATGGTCGGATTGG-3′	163
37	NQO1	Forward 5′-TCGCCGAGCAGAAGAAGATTGAAG-3′ Reverse 5′-GGTGGTGAGTGACAGCATGGC-3′	191
38	GSTA2	Forward 5′-GGAGTCAATCCGGTGGCTGTTAG-3′ Reverse 5′-GGCTCTGCTCTGCACCATCTTC-3′	163
39	ATG5	Forward 5′-GGACGCATACCAACCTGCTT Reverse 5′-TGCCATTTCAGTGGCGTACC	200
40	Dynein	Forward 5′-CGTTGCCAGCGTTACACCTATCC Reverse 5′-GCCAGGACTGCCACCAACAC	163
41	F-actin	Forward 5′-ACCTGGATTGGAGAGGATGTCAGC Reverse 5′-CGGCCTTCTTCAGCTCGTTCTTG	160
42	α-Tubulin	Forward 5′-GCGGCACGGCAAGTACATGG Reverse 5′-CTTGGTCTTGATGGTGGCGATGG	94
43	TGFβ1	Forward 5′-GCCGACACGCAGTACACCAAG Reverse 5′-GCAGGCACGGACCACCATATTG	168
44	β-actin	Forward 5′-CAACACAGTGCTGTCTGGTGGTAC-3′ Reverse 5′-CTCCTGCTTGCTGATCCACATCTG-3′	199

### Western blotting

Western blotting was performed based on a previous procedure with some modifications [28]. In brief, protein samples were separated with SDS-PAGE (8–12%) and transferred to PVDF membranes (Pall Corporation, USA). After blocking the membranes with 5% skim milk for 1 h at room temperature, the membranes were washed three times with TBST and incubated with primary antibodies, including Nrf2 (1:500, Bioss Antibodies Inc. Beijing, China), HO-1 (1:800, Bioss Antibodies Inc. Beijing), Caspase-3 (1:500, Wanleibio Co., Shanghai, China), Caspase-9 (1:1000, Wanleibio Co.), Bcl2-associated X protein (Bax) (1:500, Wanleibio Co.), Cytochrome-C (1:800, Wanleibio Co.), B cell lymphoma-2 (Bcl-2) (1:500, Wanleibio Co.) and β-actin (1:1000, Bioss Antibodies Inc. Beijing), overnight at 4 °C. The membranes were washed an additional three times with TBST and incubated with secondary goat anti-rabbit (1:3000, Bioss Antibodies Inc. Beijing) or anti-mouse (1:3000, Bioss Antibodies Inc. Beijing) horseradish peroxidase-conjugated IgG. The membranes were then visualized with enhanced chemiluminescence (ECL, Biosharp Life Sciences, China) reagent after washing three times with TBST for 10 min each time. β-actin was used as a loading control antibody.

### Statistical analysis

Statistical significance (*p* ≤ 0.05) was determined by one-way ANOVA among groups using the software statistical package for social sciences (SPSS, version 21.0). GraphPad Prism version 6.0 was used to draw all the graphs. The heatmap was made with heatmap illustrator software (HemI, version 1.0.3.7).

## Results

### Effect of baicalin on antioxidant activities

**Figure 1:**
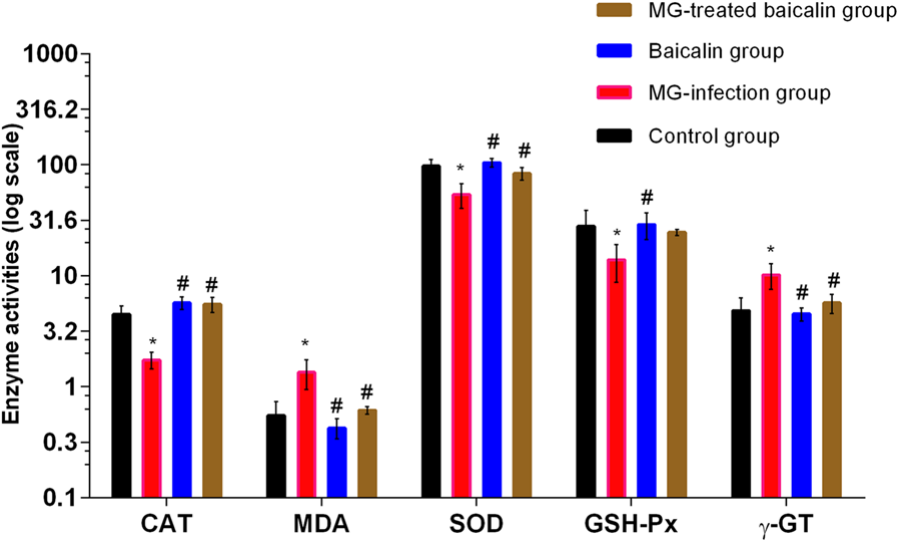
**Effect of baicalin and MG infection on chicken thymus antioxidant activities.** Antioxidant activities are displayed in this figure. The experimental groups included the control group, MG-infected group, Baicalin group (450 mg/kg) and MG-treated baicalin group (450 mg/kg). *p < 0.05 vs. the control group, and ^#^*p* < 0.05 vs. the MG-infection group. The results are expressed as the mean ± SD (*n* = 3).

The activities of antioxidants, including SOD, GSH-Px, CAT, MDA and γ-GT, (Figure 1) were examined in thymus samples of chickens from all experimental groups. CAT, SOD and GSH-Px activities were significantly (*p* < 0.05) decreased in the MG-infected group compared to the control group and the baicalin group. Meanwhile, MDA and γ-GT activities were significantly (*p* < 0.05) increased in the MG-infected group. However, baicalin treatment significantly (*p* < 0.05) alleviated the alteration in SOD, CAT, MDA and γ-GT activities, but not the alteration in GSH-Px activity, which did not show a significant increase (*p* > 0.05) in the MG-treated baicalin group compared to the MG-infection group. These findings showed that baicalin has the potential to alleviate oxidative stress in the thymus tissues of chickens. In addition, compared to the control condition, baicalin treatment alone had no significant effect on SOD, GSH-Px, CAT, MDA and γ-GT activities.

### Histopathological and ultrastructural observations

**Figure 2:**
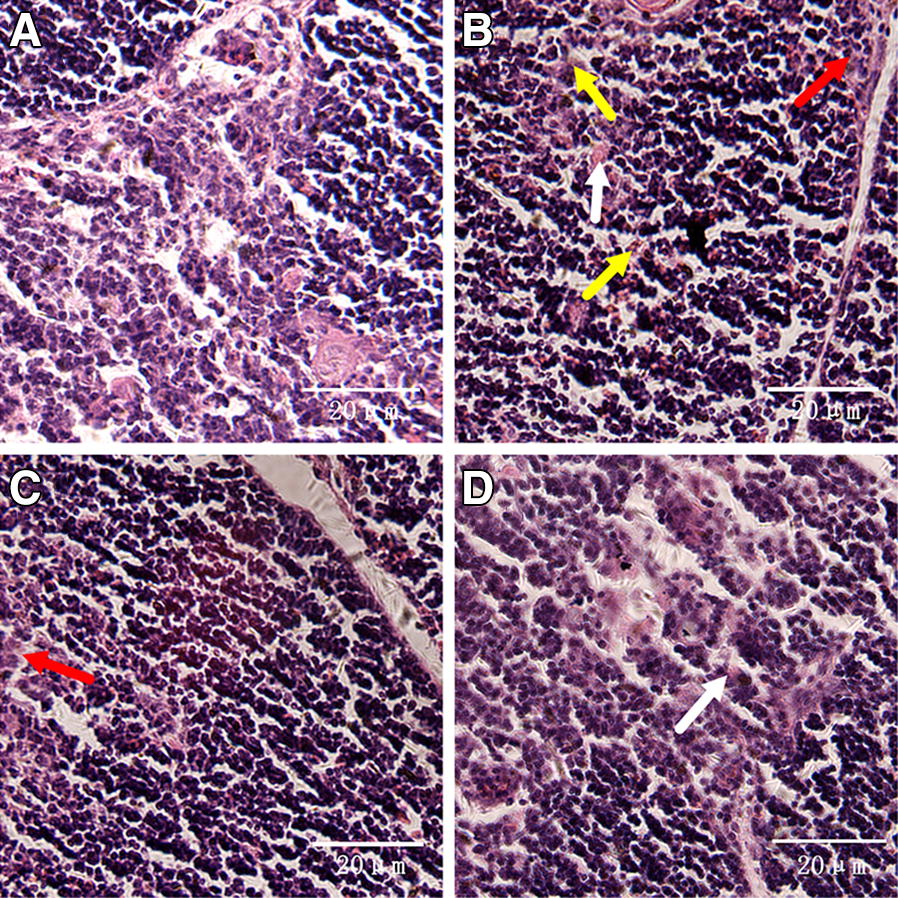
**Histopathological analysis of chicken thymus.** Histopathological examination of the thymus specimens of four experimental groups is shown in this figure (scale bar = 20 mm). The experimental groups were the **A** control group, **B** MG-infected group, **C** baicalin group (450 mg/kg), and **D** MG-treated baicalin group (450 mg/kg). The photomicrographs showed obvious reticular cells (red arrows), lymphocyte exudation (white arrows) and cell necrotic debris (yellow arrows).

**Figure 3:**
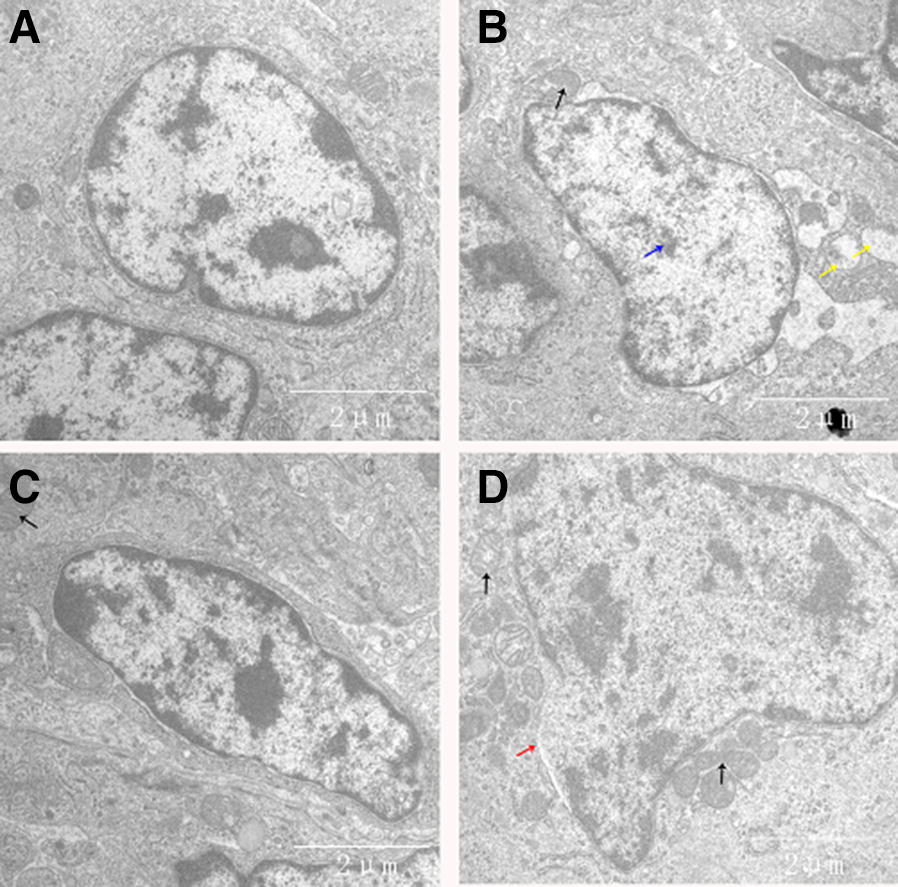
**Ultrastructural analysis of chicken thymus.** Transmission electron microscopic examination of thymus samples from the four experimental groups is shown in this figure (scale bar = 2 µm). The experimental groups were the **A** control group, **B** MG-infected group, **C** baicalin group (450 mg/kg) and **D** MG-treated baicalin group (450 mg/kg). Clear signs of apoptosis were observed, including mitochondrial swelling (black arrows), membrane deformation (blue arrows) and broken mitochondrial cristae (yellow arrows).

To clarify the protective effects of baicalin against MG infection-induced damage in the thymus, histopathological and ultrastructural observations were performed. Histopathological results (Figure 2) revealed increased nuclear debris, inflammatory cell infiltrates, and a few vacuoles in the thymocytes of chickens from the MG-infected group compared to those from the control group and baicalin group. In contrast, cells from the control group showed normal morphology, fewer vacuoles and compactly arranged cells. Ultrastructural examination (Figure 3) also showed normal morphology of cells in the control group but revealed many apoptotic features, such as chromatin condensation, mitochondrial swelling and cell damage, in the MG-infected group. However, the above abnormal morphological signs partially disappeared with baicalin treatment. These findings suggested that baicalin could protect structural integrity and inhibit MG infection-induced immune impairment in chicken thymus.

### Effect of baicalin on cytokine activities

**Figure 4:**
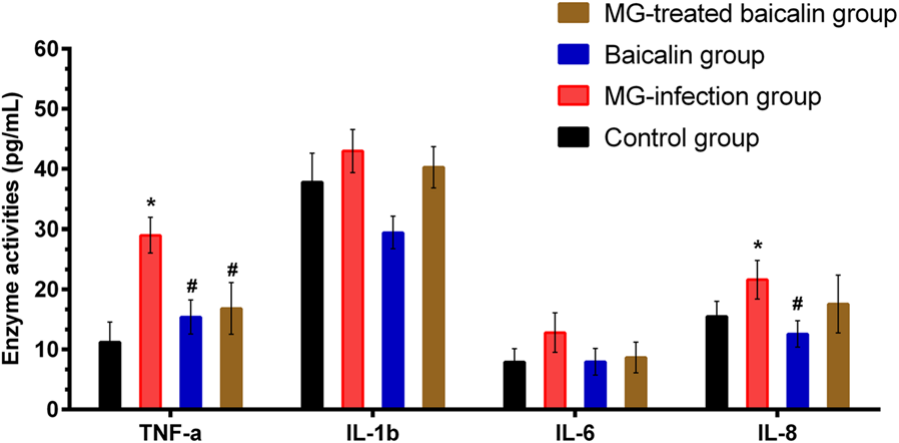
**Effect of baicalin and MG infection on Pro-inflammatory cytokine activities.** Pro-inflammatory cytokine activities are shown in this figure. The experimental groups included the control group, MG-infected group, baicalin group (450 mg/kg) and MG-treated baicalin group (450 mg/kg). *p < 0.05 vs. the control group, and ^#^*p* < 0.05 vs. the MG-infection group. The results are expressed as the mean ± SD (*n* = 3).

MG infection induced oxidative stress in the chicken thymus, which acts as a second messenger to interrupt cytokines. The dysregulation of cytokine activities caused immune impairment during MG infection [3]. ELISA was used to measure cytokine activities (Figure 4) to determine MG-induced alterations and the effect of baicalin on these activities in the thymus of chickens. Compared to the control group and the baicalin group, the MG-infected group showed increased TNF-α (*p* < 0.05), IL-6 (*p* > 0.05), IL-1β (*p* > 0.05) and IL-8 (*p* > 0.05) activities. Meanwhile, baicalin treatment significantly restored the MG-induced increase in TNF-α (*p* < 0.05) enzyme activity, but the IL-6, IL-1β and IL-8 levels were not significantly different (*p* > 0.05) between the MG-treated baicalin group and the MG-infected group. Additionally, compared to the control condition, baicalin treatment alone had no significant effect on the activities of TNF-α, IL-6, IL-1β and IL-8.

### The mRNA expression profile of genes involved in apoptosis, autophagy, inflammatory response and defence pathways in the thymus of chickens

**Figure 5:**
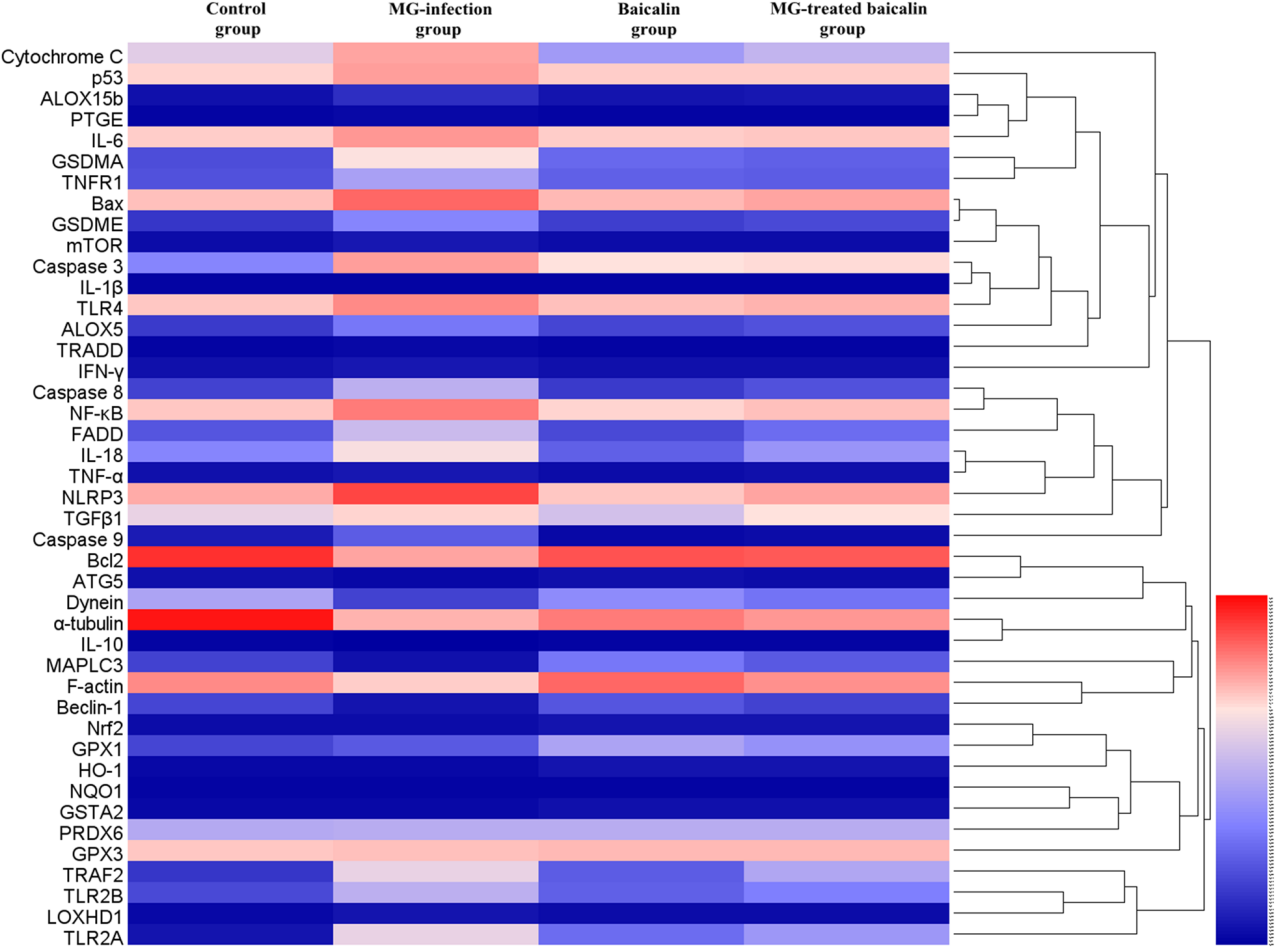
**Heat map showing the relative mRNA expression levels of genes involved in multiple signalling pathways, including apoptosis, autophagy, Nrf2, inflammation and death receptor pathways.** The mRNA expression levels of genes are shown using the indicated pseudo colour scale. The experimental groups included the control group, MG-infected group, baicalin group (450 mg/kg) and MG-treated baicalin group (450 mg/kg).

A heatmap (Figure 5) shows the mRNA expression profile of genes involved in different signalling pathways, including autophagy, apoptosis, death receptor signalling, inflammation and the Nrf2 defence pathway, in the thymus tissues of chickens. The results revealed significant changes in the mRNA expression profiles of these genes in the MG-infected group relative to the control group. However, baicalin treatment restored the changes in the mRNA expression of these genes to some extent. A detailed description of the apoptosis and Nrf2 pathways is given below.

### Baicalin inhibited MG-induced apoptosis in the thymus tissues of chickens

**Figure 6:**
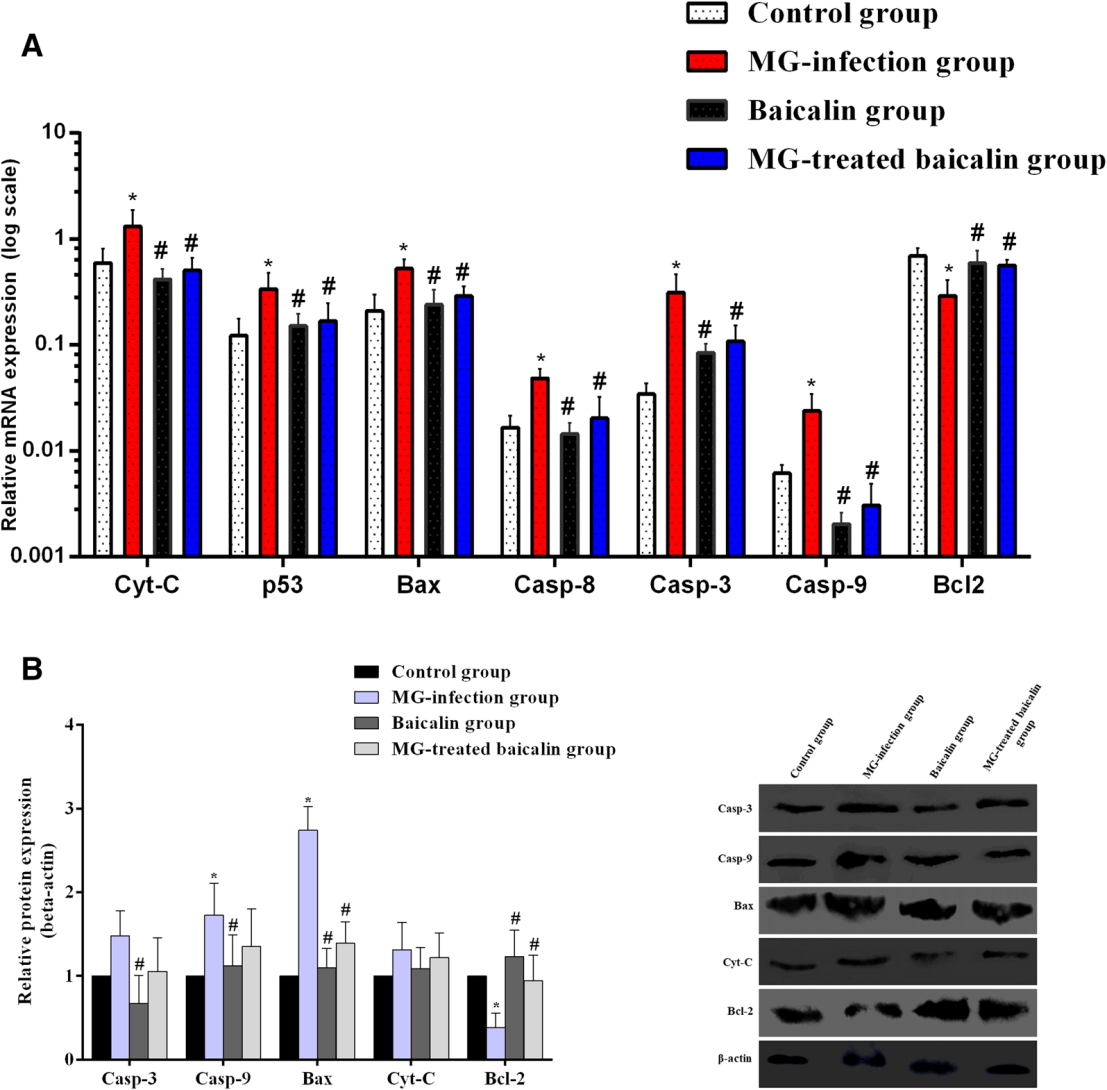
**Effect of baicalin and MG infection on apoptosis-related genes.** This Figure shows the **A** mRNA expression and **B** protein expression of apoptosis-related genes in the thymus tissues of the four experimental groups. The experimental groups included the control group, MG-infected group, baicalin group (450 mg/kg) and MG-treated baicalin group (450 mg/kg). *p < 0.05 vs. the control group, and ^#^*p* < 0.05 vs. the MG-infection group. The results are expressed as the mean ± SD (*n* = 3).

**Figure 7:**
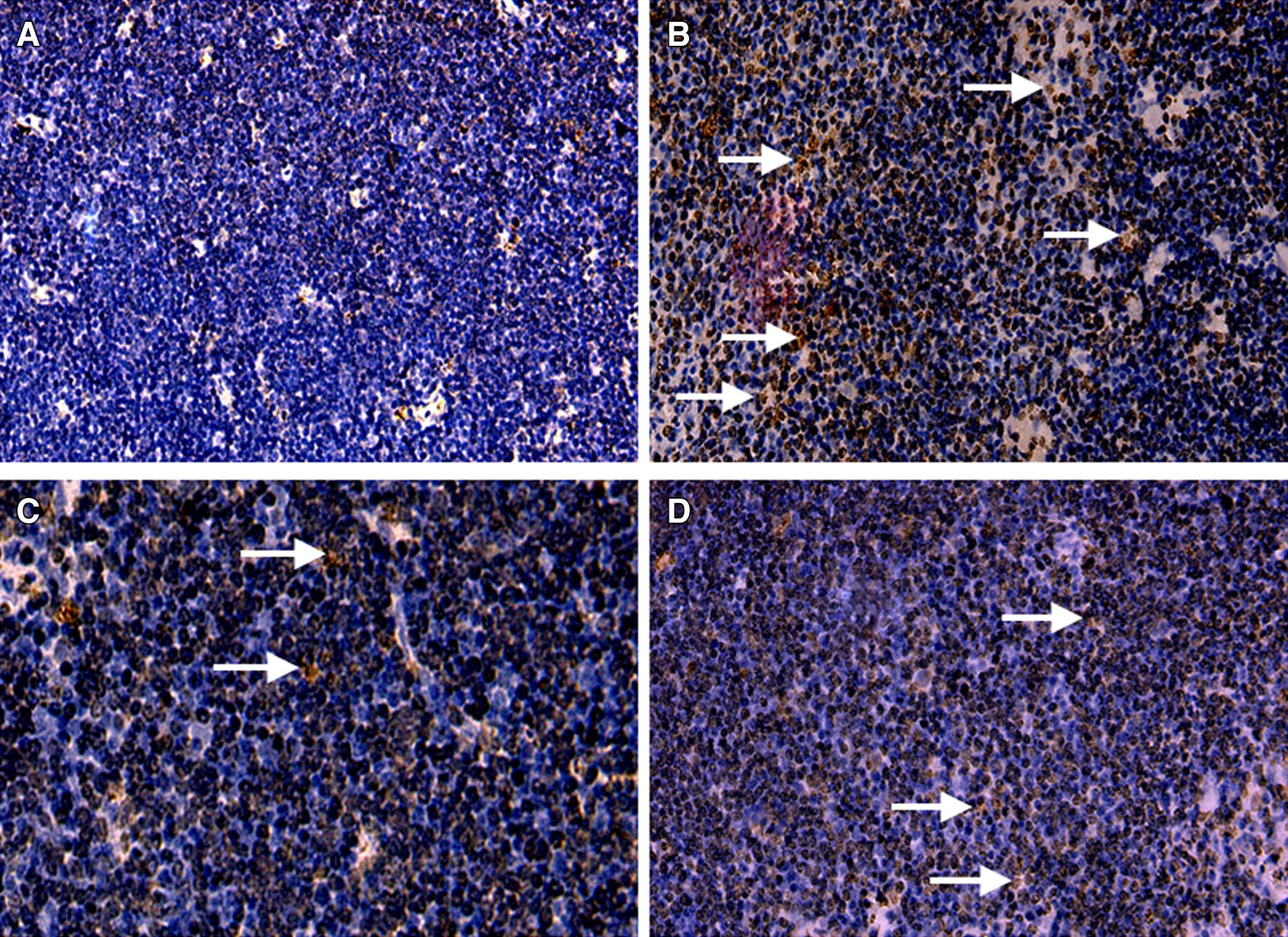
**Detection of apoptosis by TUNEL assay.** Apoptosis was analysed by TUNEL assay in all experimental groups, as shown in this figure. Brown-stained nuclei (apoptotic cells) were observed in chicken thymus. The experimental groups included the **A** control group, **B** MG-infected group, **C** baicalin group (450 mg/kg) and **D** MG-treated baicalin group (450 mg/kg).

Apoptosis plays a crucial role in the selection of T cells in the thymus. Figures 6A and B show the mRNA and protein expression profiles of apoptosis-related genes. The mRNA expression levels of Cytochrome-C, p53, Bax, Caspase-8, Caspase-3 and Caspase-9 were significantly (*p* < 0.05) enhanced in the MG-infected group, while the anti-apoptotic gene Bcl-2 was significantly (*p* < 0.05) reduced in the MG-infected group compared to the control group. In addition, caspase-9 and Bax protein expression were significantly (*p* < 0.05) enhanced in the MG-infected group. However, the increases in Caspase-3 and Cytochrome-C protein expression were not statistically significant (*p* > 0.05) in the MG-infected group compared to the control group. In contrast, Bcl2 protein levels were significantly (*p* < 0.05) reduced in the MG-infected group compared to the control group. Meanwhile, a significant (*p* < 0.05) reduction was noted in the mRNA expression of Cytochrome-C, p53, Bax, Caspase-8, Caspase-3 and Caspase-9 with baicalin intervention. Bcl2 mRNA expression was significantly (*p* < 0.05) enhanced in the MG-treated baicalin group and baicalin group compared to the MG-infected group. In addition, baicalin significantly (*p* < 0.05) alleviated the alteration in the protein expression of the caspase-9, Bax, Caspase-3 and Bcl2 genes, but not that of Cytochrome-C (*p* > 0.05). Here, we also examined apoptotic cells using a TUNEL assay (Figure 7) to determine the protective role of baicalin against MG-induced apoptosis in thymus tissues. A significant increase in apoptotic cells (positive-stained brown nuclei) was noted in the MG-infected group compared to the control group and the baicalin group. However, baicalin treatment effectively decreased the number of positively stained nuclei in the MG-treated baicalin group relative to the MG-infected group. These findings demonstrated that baicalin efficiently attenuated apoptosis and could prevent immune impairment in the thymus tissues of chickens during MG infection.

### Effect of MG infection and baicalin on the expression of the Nrf2/HO-1 pathway

**Figure 8:**
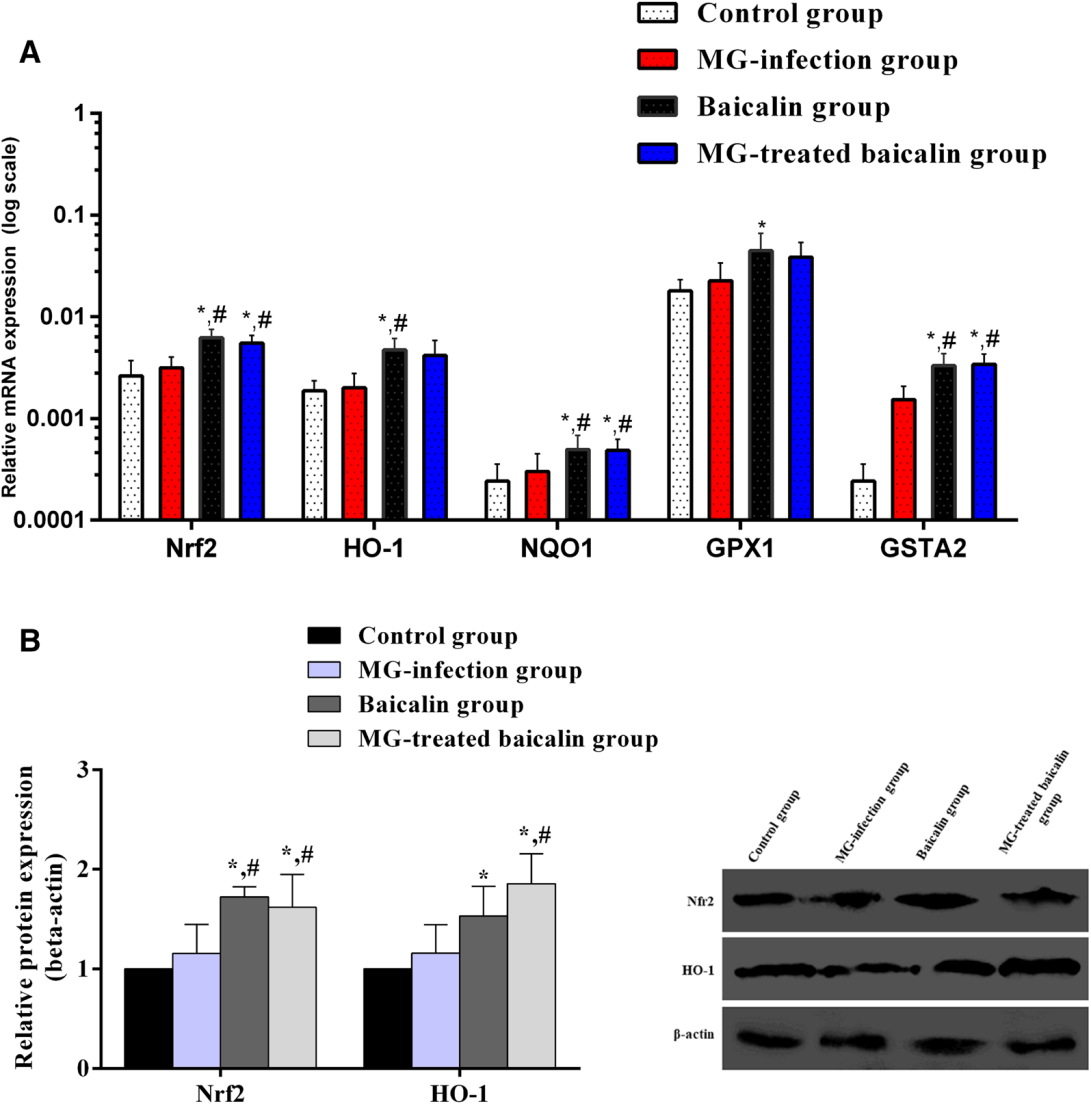
**Effect of baicalin and MG infection on the Nrf2 pathway.** This figure shows the effect of the four experimental treatments on the **A** mRNA expression and **B** protein expression levels of transcription factor Nrf2 and its downstream genes in chicken thymus tissues. The experimental groups included the control group, MG-infected group, baicalin group (450 mg/kg) and MG-treated baicalin group (450 mg/kg). *p < 0.05 vs. the control group, and ^#^*p* < 0.05 vs. the MG-infected group. The results are expressed as the mean ± SD (*n* = 3).

The Nrf2/HO-1 signalling pathway protects cells from oxidative damage and toxic insults. The mRNA and protein expression levels of the transcription factor Nrf2 and its downstream genes were examined in all experimental groups (Figure 8). Compared to the control condition, MG infection slightly increased (*p* > 0.05) the mRNA (Figure 8A) and protein expression level (Figure 8B) of Nrf2 and its downstream genes. Intriguingly, both the protein and mRNA expression levels of Nrf2 and its downstream genes were significantly (*p* < 0.05) increased in the MG-treated baicalin group compared to the control group and MG-infection group.

## Discussion

Previous studies demonstrated that MG infection causes significant economic losses to the poultry industry due to reduced egg quality and egg production, decreased carcass quality and increased susceptibility to other diseases [1]. Once infected, the birds become a carrier of this pathogen and remain infected for a long time [2]. Therefore, it is often difficult to eradicate this infection by culling entire flocks. Our data revealed that MG infection induced oxidative stress in the thymus of white leghorn chickens. Histological sections showed inflammatory cell infiltration with abnormal morphology in the MG-infected group. In addition, transmission electron microscopy results demonstrated that the structural integrity of the thymus was deteriorated and that signs of apoptosis were visible. These findings provide evidence that MG induces apoptosis in the chicken thymus. These findings are in agreement with previous studies [4] that microscopic lesions, including mild to moderate congestion and lymphocyte depletion, were prominent in the thymus of chickens infected with MG. The thymus serves a critical role in the defence mechanism of the body, providing protection and surveillance against various pathogens [29]. However, until now, MG-induced immune dysregulation in the thymus has not been reported. In the present study, MG induced apoptosis in the thymocytes of chickens. The possible cause of apoptosis is the oxidative stress produced by MG infection, which acts as a second messenger for apoptotic cell signalling and leads to lymphocyte depletion in the thymus. Thus, it is speculated that MG-induced immune impairment in chicken thymus could be due to oxidative stress damage and excessive apoptosis. Previous reports showed that oxidative stress could induce apoptosis from both mitochondrial dependent and independent pathways [30]. Additionally, oxidative stress resulted in various modifications in lipid molecules associated with damage molecular patterns leading to sterile inflammation. The molecular mechanisms of the relationship between oxidative stress and inflammation were summarized in a review [31]. Intriguingly, apoptosis is induced through pro-inflammatory mediators such as TNF-α, which plays a multifunctional role; TNF-α can also bind to the death receptor (TNF receptor 1, TNF-R1) and may directly activate apoptosis through the stimulation of caspases [32]. In the present study, a significant increase in TNF-α activity was noted in the MG-infected group. Moreover, the mRNA expression of the death receptor was obviously increased in the MG-infected group. These findings are in line with the above studies showing that oxidative stress and TNF-α are possible factors that may be involved in apoptosis and inflammation in chicken thymus. However, additional studies are needed to clarify the mechanism that relates apoptosis, inflammation and oxidative stress in chicken thymus during MG infection.

Baicalin has been reported to be a potent antioxidant, anti-inflammatory, antibacterial, anti-viral and anticancer agent [33]. The protective effects of baicalin are associated with its wide range of therapeutic properties, and baicalin can modulate various signalling pathways involved in vital body functions [18, 34]. In the present study, baicalin efficiently alleviated oxidative stress in thymus tissues, as evidenced by antioxidant activities. In addition, pro-inflammatory cytokine activities were decreased in the MG-treated baicalin group relative to the MG-infected group. These results are similar to previous studies showing that baicalin acts as a potential antioxidant and anti-inflammatory agent [20, 35, 36]. Moreover, Yang et al. demonstrated that baicalin promoted regulatory T cell differentiation and can be used for the treatment of autoimmune and inflammatory diseases [37]. In another study, baicalin inhibited the apoptosis of CD3^+^ T cells in the thymus of mice with polymicrobial sepsis [38]. Our data showed that baicalin significantly reduced MG-induced apoptosis in thymus tissues. It is worth mentioning that treatment with baicalin alone had no significant effect on the level of apoptosis in the thymus of chickens. However, additional studies are needed to investigate the effect of baicalin on the role of apoptosis in normal thymocyte differentiation in vivo. In addition to apoptosis, autophagy plays a crucial role in innate immunity and in defending against invading pathogens by presenting degraded molecules extracellularly and/or directly degrading bacterial proteins [15, 39]. A previous study demonstrated that autophagy clears *Pseudomonas aeruginosa* infection by alveolar macrophages [14]. Zhu et al. showed that baicalin inhibited influenza A virus H3N2-induced autophagy, which may contribute to its anti-viral activity [40]. In contrast, the present data revealed that baicalin activated the MG infection-mediated inhibition of autophagy. The mRNA expression of autophagy-related genes was significantly upregulated in the baicalin-treated group relative to the MG-infection group. Thus, it is speculated that autophagy may be a potential marker for the preparation and development of novel drugs against bacterial infections. In addition to targeting autophagy, the transcription factor Nrf2 regulates numerous cytoprotective genes, which are of interest to scientists and are key targets of many therapeutic agents in various ailments [20]. In the present study, the mRNA and protein expression levels of Nrf2 and its downstream genes were significantly increased in the MG-treated baicalin group compared to the MG-infected group. These results revealed that baicalin prevents oxidative stress and apoptosis in part due to its effect on the Nrf2 defence pathway. Nevertheless, additional studies are needed to determine the regulatory mechanism of baicalin on the Nrf2 pathway and apoptosis. In addition, a better understanding of the crosstalk among Nrf2, oxidative stress generation and apoptosis-related mechanisms is needed to lay a foundation for the prevention of MG infection.

In conclusion, baicalin potentially inhibited oxidative stress, pro-inflammatory cytokine activities and apoptosis and maintained the structural integrity of thymus tissues. The present study provides novel insights into the antibacterial activities of baicalin, which may be helpful in the future to investigate the relationship between multiple signalling pathways and herbal drugs against bacterial infection.

## References

[1] Ley DH, Saif YM, Barnes HJ, Gilson JR, Fadly AM, McDougald LR, Swayne DE, Editorial Board for the American Association of Avian Pathologists (2003). *Mycoplasma gallisepticum* infection. Diseases of poultry.

[2] Levisohn S, Kleven SH (2000). Avian mycoplasmosis (*Mycoplasma gallisepticum*). Rev Sci Tech.

[3] Beaudet J, Tulman ER, Pflaum K, Liao X, Kutish GF, Szczepanek SM, Silbart LK, Geary SJ (2017). Transcriptional profiling of the chicken tracheal response to virulent *Mycoplasma gallisepticum* strain R_low_. Infect Immun.

[4] Manafi M, Pirany N, Noor Ali M, Hedayati M, Khalaji S, Yari M (2015). Experimental pathology of T-2 toxicosis and mycoplasma infection on performance and hepaticfunctions of broiler chickens. Poult Sci.

[5] Savino W (2006). The thymus is a common target organ in infectious diseases. PLoS Pathog.

[6] Raviola E, Karnovsky MJ (1972). Evidence for a blood-thymus barrier using electronopaque tracers. J Exp Med.

[7] Gaunson JE, Philip CJ, Whithear KG, Browning GF (2000). Lymphocytic infiltration in the chicken trachea in response to *Mycoplasma gallisepticum* infection. Microbiology.

[8] Majumder S, Silbart LK (2016). Interaction of *Mycoplasma gallisepticum* with chicken tracheal epithelial cells contributes to macrophage chemotaxis and activation. Infect Immun.

[9] Gostner JM, Becker K, Ueberall F, Fuchs D (2015). The good and bad of antioxidant foods: an immunological perspective. Food Chem Toxicol.

[10] Li WJ, Nie SP, Peng XP, Liu XZ, Li C, Chen Y, Xie MY (2012). Ganoderma atrum polysaccharide improves age-related oxidative stress and immune impairment in mice. J Agric Food Chem.

[11] Lessard M, Savard C, Deschene K, Lauzon K, Pinilla VA, Gagnon CA, Lapointe J, Guay F, Chorfi Y (2015). Impact of deoxynivalenol (DON) contaminated feed on intestinal integrity and immune response in swine. Food Chem Toxicol.

[12] Borish LC, Steinke JW (2003). Cytokines and chemokines. J Allergy Clin Immunol.

[13] Harrison L, Brown C, Afonso C, Zhang J, Susta L (2011). Early occurrence of apoptosis in lymphoid tissues from chickens infected with strains of Newcastle disease virus of varying virulence. J Comp Pathol.

[14] Cuervo AM (2004). Autophagy: in sickness and in health. Trends Cell Biol.

[15] Yuan K, Huang C, Fox J, Laturnus D, Carlson E, Zhang B, Yin Q, Gao H, Wu M (2012). Autophagy plays an essential role in the clearance of Pseudomonas aeruginosa by alveolar macrophages. J Cell Sci.

[16] Lu Z, Xie D, Chen Y, Tian E, Muhammad I, Chen X, Miao Y, Hu W, Wu Z, Ni H, Xin J, Li Y, Li J (2017). TLR2 mediates autophagy through ERK signaling pathway in *Mycoplasma gallisepticum*-infected RAW264.7 cells. Mol Immunol.

[17] Zhao Q, Chen XY, Martin C (2016). *Scutellaria baicalensis*, the golden herb from the garden of Chinese medicinal plants. Sci Bull (Beijing).

[18] Gong WY, Zhao ZX, Liu BJ, Lu LW, Dong JC (2017). Exploring the chemopreventive properties and perspectives of baicalin and its aglyconebaicalein in solid tumors. Eur J Med Chem.

[19] Shen K, Feng X, Pan H, Zhang F, Xie H, Zheng S (2017). Baicalin ameliorates experimental liver cholestasis in mice by modulation of oxidative stress, inflammation, and NRF2 transcription factor. Oxid Med Cell Longev.

[20] Shi L, Hao Z, Zhang S, Wei M, Lu B, Wang Z, Ji L (2018). Baicalein and baicalin alleviate acetaminophen-induced liver injury by activating Nrf2 antioxidative pathway: the involvement of ERK1/2 and PKC. Biochem Pharmacol.

[21] Bajpai VK, Alam MB, Ju MK, Kwon KR, Huh YS, Han YK, Lee SH (2018). Antioxidant mechanism of polyphenol-rich Nymphaea nouchali leaf extract protecting DNA damage and attenuating oxidative stress-induced cell death via Nrf2-mediated heme-oxygenase-1 induction coupled with ERK/p38 signaling pathway. Biomed Pharmacother.

[22] Xiao X, Zhao DH, Yang X, Shi W, Deng H, Ma J, Zhang S, Liu YH (2014). *Mycoplasma gallisepticum* and *Escherichia coli* mixed infection model in broiler chickens forstudying valnemulin pharmacokinetics. J Vet Pharmacol Ther.

[23] Cheng P, Wang T, Li W, Muhammad I, Wang H, Sun X, Yang Y, Li J, Xiao T, Zhang X (2017). Baicalin alleviates lipopolysaccharide-induced liver inflammation in chicken by suppressing TLR4-mediated NF-κB pathway. Front Pharmacol.

[24] Muhammad I, Wang H, Sun X, Wang X, Han M, Lu Z, Cheng P, Hussain MA, Zhang X (2018). Dual role of dietary curcumin through attenuating AFB_1_-induced oxidative stress and liver injury via modulating liver phase-I and phase-II enzymes involved in AFB1 bioactivation and detoxification. Front Pharmacol.

[25] Wang X, Muhammad I, Sun X, Han M, Hamid S, Zhang X (2018). Protective role of curcumin in ameliorating AFB1-induced apoptosis via mitochondrial pathway in liver cells. Mol Biol Rep.

[26] Wang J, Yi M, Chen X, Muhammad I, Liu F, Li R, Li J, Li J (2016). Effects of colistin on amino acid neurotransmitters and blood-brain barrier in the mouse brain. Neurotoxicol Teratol.

[27] Livak KJ, Schmittgen TD (2001). Analysis of relative gene expression data using real time quantitative PCR and the 2 (-Delta Delta C (T)) method. Methods.

[28] Wang H, Muhammad I, Li W, Sun X, Cheng P, Zhang X (2018). Sensitivity of Arbor Acres broilers and chemoprevention of aflatoxin B_1_-induced liver injury by curcumin, a natural potent inducer of phase-II enzymes and Nrf2. Environ Toxicol Pharmacol.

[29] Thapa P, Farber DL (2019). The role of the thymus in the immune response. Thorac Surg Clin.

[30] Sinha K, Das J, Pal PB, Sil PC (2013). Oxidative stress: the mitochondria-dependent and mitochondriaindependent pathways of apoptosis. Arch Toxicol.

[31] Lugrin J, Rosenblatt-Velin N, Parapanov R, Liaudet L (2014). The role of oxidative stress during inflammatory processes. Biol Chem.

[32] Linkermann A, Stockwell BR, Krautwald S, Anders HJ (2014). Regulated cell death and inflammation: an auto-amplification loop causes organ failure. Nat Rev Immunol.

[33] Xiao JR, Do CW, To CH (2014). To, Potential therapeutic effects of baicalein, baicalin, and wogonin in ocular disorders. J Ocul Pharmacol Ther.

[34] Yang X, Yang J, Zou H (2013). Baicalin inhibits IL-17-mediated joint inflammation in murine adjuvant-induced arthritis. Clin Dev Immunol.

[35] Dinda B, Dinda S, DasSharma S, Banik R, Chakraborty A, Dinda M (2017). Therapeutic potentials of baicalin and its aglycone, baicalein against inflammatory disorders. Eur J Med Chem.

[36] Wen YF, Zhao JQ, Bhadauria M, Nirala SK (2013). Baicalin prevents cadmium induced hepatic cytotoxicity oxidative stress and histomorphometric alternations. Exp Toxicol Pathol.

[37] Yang J, Yang X, Li M (2012). Baicalin, a natural compound, promotes regulatory T cell differentiation. BMC Complement Altern Med.

[38] Zhu J, Wang J, Sheng Y, Zou Y, Bo L, Wang F, Lou J, Fan X, Bao R, Wu Y, Chen F, Deng X, Li J (2012). Baicalin improves survival in a murine model of polymicrobial sepsis via suppressing inflammatory response and lymphocyte apoptosis. PLoS One.

[39] Law AH, Lee DC, Yuen KY, Peiris M, Lau AS (2010). Cellular response to influenza virus infection: a potential role for autophagy in CXCL10 and interferon-alpha induction. Cell Mol Immunol.

[40] Zhu HY, Han L, Shi XL, Wang BL, Huang H, Wang X, Chen DF, Ju DW, Feng MQ (2015). Baicalin inhibits autophagy induced by influenza A virus H3N2. Antivir Res.

